# The influence of face mask on social spaces depends on the behavioral immune system

**DOI:** 10.3389/fnins.2022.991578

**Published:** 2022-11-11

**Authors:** Laurie Geers, Yann Coello

**Affiliations:** Univ. Lille, CNRS, UMR 9193 - SCALab - Sciences Cognitives et Sciences Affectives, Lille, France

**Keywords:** social interaction, reachable space, perceived vulnerability to disease, interpersonal distance, comfort distance judgment, reachability judgment, COVID-19

## Abstract

Interacting with objects and people requires specifying localized spaces where these interactions can take place. Previous studies suggest that the space for interacting with objects (i.e., the peripersonal space) contributes to defining the space for interacting with people (i.e., personal and interpersonal spaces). Furthermore, situational factors, such as wearing a face mask, have been shown to influence social spaces, but how they influence the relation between action and social spaces and are modulated by individual factors is still not well understood. In this context, the present study investigated the relationship between action peripersonal and social personal and interpersonal spaces in participants approached by male and female virtual characters wearing or not wearing a face mask. We also measured individual factors related to the behavioral immune system, namely willingness to take risks, perceived infectability and germ aversion. The results showed that compared to peripersonal space, personal space was smaller and interpersonal space was larger, but the three spaces were positively correlated. All spaces were altered by gender, being shorter when participants faced female characters. Personal and interpersonal spaces were reduced with virtual characters wearing a face mask, especially in participants highly aversive to risks and germs. Altogether, these findings suggest that the regulation of the social spaces depends on the representation of action peripersonal space, but with an extra margin that is modulated by situational and personal factors in relation to the behavioral immune system.

## Introduction

In order to act on objects in the surroundings, the visual and sensorimotor systems must combine their representations of the environment and the body to define an arm-reach space, classically defined as the peripersonal space (PPS, Rizzolatti et al., 1981; Coello and Iachini, 2015, 2021; di Pellegrino and Làdavas, 2015). Based on electrophysiological studies of the monkey brain, the particular aspect of PPS representation was originally pinpointed by Rizzolatti et al. (1981). It was conceived as an interface between the body and the environment contributing to the orientation of attention toward objects that represent potential targets for motor actions, and would thereby serve two essential functions: selecting potential actions toward incentive objects and protecting the body from threatening objects (Fogassi et al., 1996; Graziano and Cooke, 2006; Brozzoli et al., 2011; Coello and Cartaud, 2021). A particularity is that stimuli located in the PPS are coded through multisensory processes (e.g., Rizzolatti et al., 1981; di Pellegrino et al., 1997; Pavani et al., 2000; Makin et al., 2007; Serino et al., 2015), which allow an enhanced perceptual and cognitive processing of those stimuli, and prepare the motor systems to interact with them (Gori et al., 2011; Belardinelli et al., 2018; Blini et al., 2018). This enhanced processing is thought to subtend the selection of approach-avoidance behavior depending on the readiness of the body to interact with appetitive or aversive stimuli (Corr, 2013; Coello et al., 2018; Gigliotti et al., 2021). Accordingly, brain-imaging and brain stimulation studies revealed that objects processing in PPS recruits not only the sensory brain areas (e.g., visual, auditory, and olfactory), but also the sensorimotor areas including the posterior parietal and ventral premotor cortices (Grafton et al., 1997; Chao and Martin, 2000; Cardellicchio et al., 2011; Proverbio, 2012; Bartolo et al., 2014; Wamain et al., 2016). Altogether, these findings support the idea that PPS is an action space represented on the basis of motor information similarly to action execution or observation (Babiloni et al., 1999; Binkofski et al., 1999; Làvadas and Serino, 2008; Medendorp et al., 2011; Finisguerra et al., 2015).

Daily interaction with the environment also implies social stimuli. One key component of social interaction is the regulation of the distance one maintains with others (Hediger, 1950, 1968; Hall, 1966; Coello and Cartaud, 2021). Indeed, early research in ethology revealed that all animals maintain a certain distance from each other in ecological conditions, both within and between species (Hediger, 1950, 1968). Based on these observations, the social psychologist Hall (1966) suggested that every human being is surrounded by a series of bubbles that serve to maintain proper spacing between individuals in a social context, suggesting that inter-individual distances constitute the foundation of natural social interactions. Accordingly, if the inter-individual distance is too wide, it is not suitable for natural social interactions, and if it is too narrow, and thereby violates personal space (PS), it generates discomfort (Sommer, 1959; Hayduk, 1978; Kennedy et al., 2009; Lloyd, 2009). The efficient inter-individual distance or interpersonal space (IPS) thus results from the subtle balance between the need to interact efficiently and a variety of other factors that are driven by approach-avoidance motivations (Argyle and Dean, 1965).

Beyond facilitating social interactions, inter-individual distance regulation seems to be rooted in sensorimotor representations. Indeed, a number of experiments revealed that PS (i.e., the space immediately surrounding the body that cannot be intruded by others without causing discomfort) was related to PPS representation. As evidence, Iachini et al. (2014, 2016) found both spaces to have a similar size and be commonly affected by the nature, age, and gender of the facing stimulus, with spaces being reduced with humans as compared to robots and cylinders, with females as compared to males, and with children as compared to adults. In another study, Iachini et al. (2015) further showed that both spaces were positively correlated to anxiety. These behavioral results were further corroborated by a brain imaging study showing that the frontoparietal areas known to be involved in PPS representation were also activated by PS intrusions (Vieira et al., 2020; in addition to subcortical areas associated with emotion regulation; Kennedy et al., 2009). This spatial coherence between action and social spaces suggested that they share common motor processes (Lloyd, 2009; Coello and Iachini, 2015, 2021). In particular, it has been proposed that the sensorimotor processes of PPS serve as a spatial reference to define social spaces (Coello and Cartaud, 2021). As evidence, Quesque et al. (2017) showed that extending arm length’s representation through tool-use increased not only PPS (Canzoneri et al., 2013; Bourgeois et al., 2014) but also PS. However, Patané et al. (2016, 2017) found dissociated effects of tool-use on PPS and PS, suggesting there is no functional overlap between the two spaces. Moreover, most studies investigated the smallest inter-individual distance that is tolerated (PS), leaving aside the inter-individual one would actually maintain (IPS). Hence, the link between action and social spaces, in particular PS and IPS, is still debated and remains to be further investigated.

The COVID-19 pandemic began in China in the fall of 2019 and quickly spread internationally, with today the death toll of more than 521 million people infected and nearly 6.5 million deaths across the world (WHO Health Emergency Dashboard Homepage, May 2022). To slow down the pandemic, governments have taken drastic measures to quickly find a vaccine, but also to adapt human behavior to prevent contamination. In accordance with WHO guidelines, most governments have mandated the use of barrier gestures in social contexts such as regular hand-washing, maintaining an inter-individual distance of 1–2 m, and wearing a medical face mask. Although highly encouraged due to its obvious sanitary impact, wearing a face mask was not immune to social consequences that have only begun to be studied scientifically in the last 2 years, and its interaction with other barrier gestures such as social distancing is still not well understood (Najmi et al., 2021). The earliest study that was performed (i.e., at the end of the first French lockdown period; March–May 2020) showed that PS was much shorter when facing someone wearing a face mask than someone without a face mask (Cartaud et al., 2020). This effect, associated with a higher feeling of trustworthiness, was confirmed in a number of following studies and extended to IPS (Iachini et al., 2021; Lisi et al., 2021; Luckman et al., 2021; Kroczek et al., 2022). Interestingly, the effect of wearing a face mask on social interactions was found to also alter facial emotion recognition (Carbon, 2020; Bani et al., 2021; Grundmann et al., 2021; Noyes et al., 2021; Cooper et al., 2022; Ramachandra and Longacre, 2022), in adults as in young children (Gori et al., 2021). However, in all these studies the effect of individual characteristics on the regulation of social spaces when interacting with people wearing a face mask was not taken into account. In this respect, the behavioral immune system (BIS; i.e., proactive behavioral mechanisms that inhibit contact with pathogens such as inference of risk of infection, germ aversion and perceived infectability) has been shown to be one of the best predictors of social space: those whose BIS was more reactive preferred to keep larger physical distances in social interactions (Hromatko et al., 2021). Besides this direct impact, the BIS may modulate the effect of face mask on social distance regulation as face mask also aims to decrease exposure to pathogens. According to the homeostatic model proposed by Coello and Cartaud (2021), social spaces are built on the PPS representation plus an extra margin that adapts as a function of the perceived valence of the social stimulus. Hence, face mask (and the associated trust) may influence social PS and IPS by reducing this margin of safety, while leaving PPS unaffected.

In the present study, we investigated the relationship between action and social spaces by requiring participants to perform reachability (probing PPS), comfort (probing IPS), and discomfort (probing PS) distance judgments while facing approaching male and female virtual characters wearing a face mask or not. We further investigated how individual factors related to the BIS, such as willingness to take risks, germ aversion and perceived infectability, modulate the effect of face mask and gender on the different spaces. Due to the shared sensorimotor underpinning of the action and social spaces, and in line with the homeostatic model, we hypothesized a positive correlation between PPS, PS, and IPS as well as a reduction of the social spaces in the presence of a social stimulus wearing a face mask. Furthermore, individuals who perceive themselves as highly infectable, averse to germs and/or are not willing to take risks were expected to perceive social stimuli as more negative (Thiebaut et al., 2021), especially those without a face mask, and therefore to show a stronger effect of face mask on social spaces.

## Materials and methods

### Participants

Forty students from the Université of Lille [France, 20 females, mean (*M*) age ± standard deviation (*SD*) = 22.4 ± 3.4 years] participated in this study. They were all right-handed and had normal or corrected-to-normal vision. A sample size analysis performed in G*Power indicated that at least 34 participants were required to observe an effect characterized by a small effect size (Cohen’s *f* = 0.15) and a high-power criterion (0.8) in a 4 × 2 × 2 repeated-measure ANOVA. The research project was approved by the Research Ethics Board of the University of Lille (CESC Lille, Ref. 2021-515-S95).

### Task and procedure

The experiment was realized in the laboratory between April and May 2022. Wearing a face mask was not mandatory in France at that time and participants did not wear a face mask during the experiment. Each participant performed three behavioral tasks in virtual reality before completing two questionnaires and evaluating the stimuli. The following behavioral tasks were performed in a counterbalanced order while standing with a response button in the dominant hand and wearing a head-mounted display:

*Comfort Distance Judgment*. The participants were required to press the response button as soon as the virtual character approaching them was judged at the most comfortable distance to interact with them. Each trial started with the appearance of a virtual character at 300 cm in front of the participants for 500 ms, which then walked toward the participants along the radial sagittal axis at a velocity of 0.75 m/s. Whenever the participants pressed the response button, the virtual character stopped moving and remained still for 1,000 ms before disappearing. The next trial started at a random delay between 800 and 850 ms following the disappearance of the previous virtual character. The task consisted of 24 trials (2 characters’ genders × 2 mask conditions × 6 repetitions) and lasted about 3 min. This judgment task was used to assess IPS.

*Discomfort Distance Judgment*. The same procedure as in the comfort distance judgment was used, except that the participants were required to press the response button as soon as the virtual character approaching them was at a distance that made them feel uncomfortable. This judgment task was used to assess PS.

*Reachability Distance Judgment*. The same procedure as in the comfort and discomfort distance judgments was used, except that the participants were required to press the response button as soon as they judged being able to tap on the shoulder of the approaching virtual character, without actually performing any movement. This judgment task was used to assess PPS.

The participants then completed the two following questionnaires:

*Willingness to take risks* [excerpt from the Socio-Economic Panel (SOEP); Goebel et al., 2019] including a question on attitude toward risk in general, and five questions on attitude toward risk in specific domains: car driving, financial matters, leisure and sports, career, trust toward strangers and health. The participants had to indicate their willingness to take risks on an 11-point scale, with 0 indicating complete unwillingness to take risks, and 10 indicating complete willingness to take risks.

*Perceived Vulnerability to Disease* (PVD; Duncan et al., 2009) consisting of two subscales: (1) Perceived Infectability (7 items), assessing beliefs about one’s vulnerability to catching infectious diseases and (2) Germ Aversion (8 items), assessing emotional discomfort in contexts that evoke pathogen transmission. Items were answered on a 7-points Likert scale ranging from “Strongly disagree” to “Strongly agree.”

Finally, the participants evaluated the emotional valence, trustworthiness and healthiness of each virtual character used in the behavioral tasks on a continuous scale ranging from 0 (“Very negative” for emotional valence, “Very untrustworthy” for trustworthiness, and “Very sick” for healthiness) to 100 (“Very positive,” “Very trustworthy” and “Very healthy”).

### Apparatus and stimuli

**FIGURE 1:**
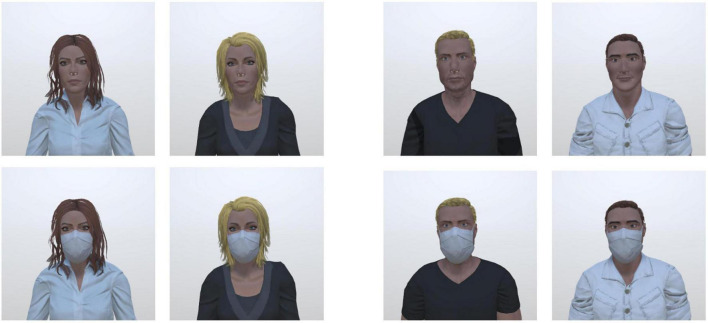
Virtual characters used in the three experimental tasks (male and female characters with a neutral facial expression wearing a face mask or not from the ATHOS database; Cartaud and Coello, 2020).

The virtual stimuli were presented, through an HTC Vive Pro head-mounted display, in a virtual room measuring 6 m × 5 m × 3 m, and consisting of a white floor, a gray ceiling and gray walls. The stimuli consisted of four human characters (two males and two females) selected from the ATHOS database (Cartaud and Coello, 2020).^1^ We adapted the hair, eye, and clothes’ color in order to match them across genders. The characters had a neutral facial expression, looked straight ahead and were presented with and without a face mask (Figure 1). The height of the stimuli was adapted so that the eye level of the virtual characters matched the eye level of the participant.

### Data analyzes

The data were analyzed using R (version 4.1.0) and R Studio software (version 1.3.1093).

#### Action and social spaces’ extent

To compute the extent of the PPS, PS, and IPS, we averaged for each participant, mask condition, and character’s gender, the distance of the visual character at the time of the response in the reachability, discomfort, and comfort distance judgments, respectively. We then compared the different spaces in terms of their average extent and their sensitivity to gender and face mask by entering the extent in a linear mixed model (LMM) including participant as a random intercept, and Space (PPS, PS vs. IPS), Gender (female vs. male), and Mask (unmasked vs. masked) as fixed effects using the *lme4* R package (Bates et al., 2011). We also planned to compute a LMM including Gender and Mask as fixed factors for each task separately in order to check whether we replicated the previously reported effect of gender on PS (Iachini et al., 2014, 2016) and of face mask on PS (Cartaud et al., 2020) and IPS (Iachini et al., 2021; Kroczek et al., 2022). In order to investigate how the effect of face mask and gender interact with individual factors, we conducted the same LMM with the score to the Risk or PVD questionnaire as an additional continuous fixed-effect. The model parameters were estimated using the Laplace approximation and were statistically tested using Wald’s *χ*^2^. Bonferroni-corrected *post-hoc* pairwise contrasts were performed using the *emmeans* package (Lenth et al., 2019).

#### Subjective evaluation of the stimuli

We verified that our different stimuli were judged as similar in terms of emotional valence, healthiness, and trustworthiness to (1) ascertain that the effect of gender was not mediated by differences in perceived valence and (2) investigate whether the effect of face mask might be mediated by differences in perceived trustworthiness or healthiness. To do so, we conducted separate repeated-measures ANOVAs on each of the subjective measure (perceived emotional valence, healthiness, and trustworthiness) with Gender (female vs. male) and Face Mask (unmasked vs. masked) as within-subject variables.

#### Correlation analysis

We then further investigated the relationship between the extent of the different spaces with pairwise correlation analyzes. We computed the Pearson *r* coefficient for each pair of spaces, gender, and face mask conditions. As the results were similar across Gender and Face mask conditions (see details in Supplementary material), we reported only the *r* coefficient for each pair of spaces averaged over Gender and Face mask conditions in the main manuscript.

## Results

### Effect of face mask and gender on action and social spaces

**FIGURE 2:**
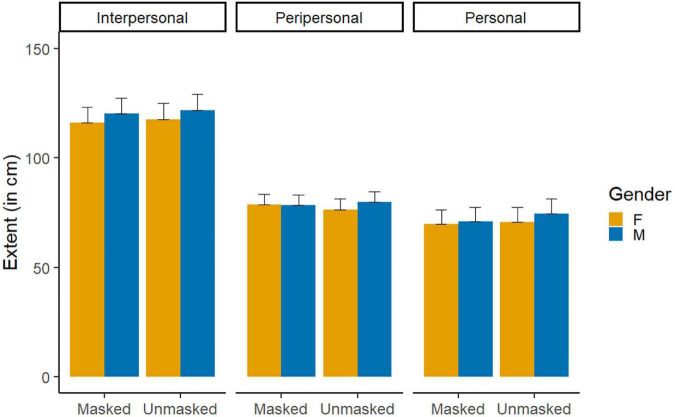
Mean interpersonal space (IPS), peripersonal space (PPS), and personal space (PS) when facing male and female characters wearing a face mask or not. Error bars represent the standard errors.

The general LMM on the extent of the different spaces only showed a significant main effect of Space, *χ*^2^(2) = 412.10, *p* < 0.001, *η*^2^ = 0.49. The average size ± standard error [*SE*] was 118.9 ± 3.6 cm for the IPS, 78.4 ± 2.4 cm for the PPS, and 71.6 ± 3.3 cm for the PS. *Post-hoc* pairwise comparisons showed that all spaces were significantly different in extent (all *p*-values <0.022; Figure 2).

*Interpersonal space*. The LMM conducted on the extent of IPS showed a main effect of Gender, *χ*^2^(1) = 10.32, *p* = 0.001, *η*^2^ = 0.08, indicating that females were preferentially placed at shorter distances (116.9 ± 5.1 cm) than males (121.00 ± 5.1 cm; Figure 2).

*Personal space*. The LMM on the extent of PS showed a main effect of Gender, *χ*^2^(1) = 5.97, *p* = 0.025, *η*^2^ = 0.05, indicating that females were tolerated closer (70.3 ± 4.6 cm) than males (72.84 ± 4.7 cm; Figure 2). There was also a significant effect of Mask, *χ*^2^(1) = 5.13, *p* = 0.023, *η*^2^ = 0.04, indicating that masked virtual characters were tolerated at a shorter distance (70.4 ± 4.6 cm) than unmasked ones (72.7 ± 4.7 cm; Figure 2).

*Peripersonal space*. The LMM showed a significant a main effect of Gender, *χ*^2^(1) = 3.96, *p* = 0.046, *η*^2^ = 0.03, embedded in a Gender × Mask interaction, *χ*^2^(1) = 5.24, *p* = 0.022, *η*^2^ = 0.04. *Post-hoc* pairwise contrasts indicated that females were judged reachable at a shorter distance than males, but only when the characters were unmasked, *t*(117) = −3.47, *p* = 0.003, and not when they were masked, *t*(117) = 0.211, *p* = 0.832 (Figure 2).

### Interaction between spaces and willingness to take risks

**FIGURE 3:**
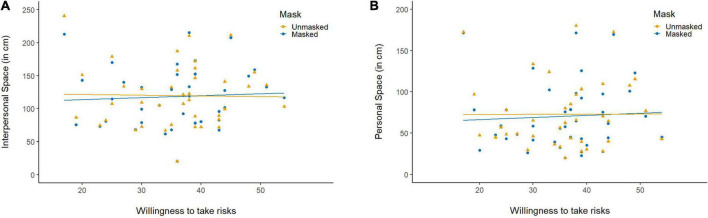
The interaction between the effect of the face mask and the willingness to take risks on **(A)** interpersonal space (IPS) and **(B)** personal space (PS).

*Interpersonal space*. The LMM conducted on the extent of IPS including Risk as a third fixed effect showed in addition to the main effect of Gender, a significant Risk × Mask interaction, *χ*^2^(1) = 7.90, *p* = 0.004, *η*^2^ = 0.06. In particular, individuals who are not willing to take risks preferred to place unmasked characters further away than masked characters, while the reverse was observed in individuals who are willing to take risks (Figure 3A).

*Personal space*. The LMM on PS showed that the effect of Mask was embedded in a significant Mask × Risk interaction, *χ*^2^(1) = 3.98, *p* = 0.046, *η*^2^ = 0.03, indicating that individuals that are not willing to take risks felt more quickly discomfortable when facing unmasked than masked characters, while there was no difference in individuals who are willing to take risks (Figure 3B).

*Peripersonal space*. The LMM showed no main effect of Risk or any interaction with the other fixed effects.

### Interaction between spaces and perceived vulnerability to disease

**FIGURE 4:**
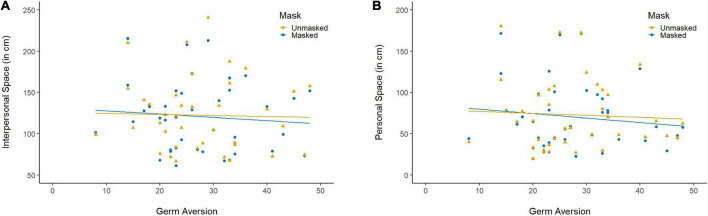
The interaction between the effect of the face mask and germ aversion on **(A)** interpersonal space (IPS) and **(B)** personal space (PS).

*Interpersonal space*. The LMM conducted on the extent of IPS including PVD as a third fixed effect showed no effect of PVD and no interaction with the other effects. However, when adding the score to the subscales rather than the total score as an additional fixed effect, there was a significant Mask × Germ Aversion interaction, *χ*^2^(1) = 3.90, *p* = 0.048, *η*^2^ = 0.03, indicating that individuals with high germ aversion preferentially placed unmasked virtual characters further away than masked virtual characters while no difference to a slight opposite trend was observed in individuals with low germ aversion (Figure 4A). There was no significant Mask × Perceived Infectability interaction, *χ*^2^(1) = 0.15, *p* = 0.694, *η*^2^ < 0.01.

*Personal space*. The LMM conducted on the extent of PS showed that the main effect of Mask was embedded in a Mask × PVD interaction, *χ*^2^(1) = 4.89, *p* = 0.027, *η*^2^ = 0.04. The interaction indicates that individuals with high scores on the PVD scale felt more quickly uncomfortable when facing unmasked virtual characters than masked virtual characters, while the reverse was observed in individuals with low PVD scores. When adding the score to the subscales rather than the total score as an additional fixed effect, there was a significant Mask × Germ Aversion interaction, *χ*^2^(1) = 7.22, *p* = 0.007, *η*^2^ = 0.06, but no Mask × Perceived Infectability interaction, *χ*^2^(1) = 0.81, *p* = 0.368, *η*^2^ = 0.00, suggesting that the effect was mainly driven by germ aversion (Figure 4B).

*Peripersonal space*. The LMM showed no effect of PVD (either when considering the total score or the score to each subscale) or any interaction with the other fixed effects.

### Subjective evaluation of the stimuli

The repeated-measure ANOVAs showed no significant effect of Gender (female vs. male) or Face mask condition (unmasked vs. masked) on perceived healthiness, *F*_*Gender*_(1, 39) = 0.62, *p*_*Gender*_ = 0.434, *η*^2^_*Gender*_ = 0.02, *F*_*Mask*_(1, 39) = 1.67, *p*_*Mask*_ = 0.203, *η*^2^_*Mask*_ = 0.04, trust, *F*_*Gender*_(1, 39) = 0.07, *p*_*Gender*_ = 0.795, *η*^2^_*Gende*_ < 0.01, *F*_*Mask*_(1, 39) = 2.75, *p*_*Mask*_ = 0.105, *η*^2^_*Mask*_ = 0.07, or perceived emotional valence, *F*_*Gender*_(1, 39) = 0.70, *p*_*Gender*_ = 0.409, *η*^2^_*Gender*_ = 0.02, *F*_*Mask*_(1, 39) = 2.68, *p*_*Mask*_ = 0.109, *η*^2^_*Mask*_ = 0.06. There was, however, a significant interaction between Face mask and Gender on the perceived emotional valence, *F*(1, 39) = 9.13, *p* = 0.004, *η*^2^_*Mask*_ = 0.19. *Post-hoc* paired *t*-test (corrected with Bonferroni) further indicated that unmasked male characters were perceived noticeably more negative (*M* ± SE = 48.84 ± 2.28) than masked male characters (53.96 ± 2.25), *t*(39) = 2.31, *p* = 0.024, while there was no difference between the masked (54.22 ± 2.43) and unmasked (56.20 ± 2.45) female characters.

### Correlation between peripersonal space, personal space, and interpersonal space

All three spaces were positively correlated to each other in all stimuli (i.e., female unmasked, female masked, male unmasked, and male masked; see details in Supplementary material). The Pearson *r* coefficients averaged over Gender and Face Mask conditions are reported in Table 1.

**TABLE 1:** Pearson correlation matrix for the average size of peripersonal space (PPS), personal space (PS) and interpersonal space (IPS).

	Peripersonal	Personal	Interpersonal
Peripersonal	1		
Personal	0.54**	1	
Interpersonal	0.57**	0.68**	1

**P-value <0.01.

## Discussion

The aim of the present study was to investigate the relationship between the action and social spaces surrounding the body by testing whether they are (1) correlated to each other and (2) similarly impacted by gender and face mask. We further investigated whether individual differences in the BIS modulate the effect of face mask on the different spaces. Our prediction was that if the sensorimotor processes of PPS contribute to the regulation of the social spaces, all three spaces should be positively correlated and be similarly impacted by gender and face mask. Furthermore, the effect of face mask was expected to influence mainly PS and IPS, and to be stronger in individuals with a reactive BIS, and thus with high perceived infectability and high aversion to risks and germs.

**FIGURE 5:**
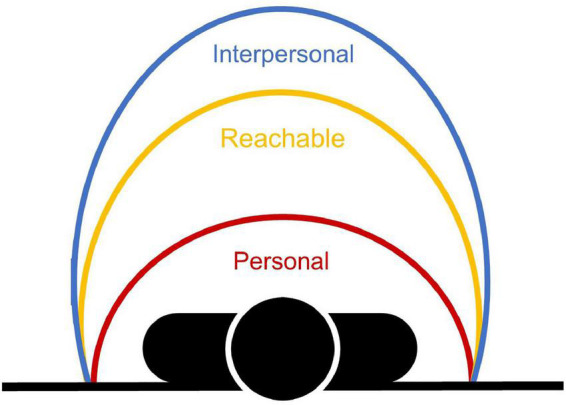
Schematic representation of the organization of the different spaces.

The results showed that compared to the PPS (78 cm), PS was smaller (72 cm) and IPS was larger (119 cm). This means that participants preferred placing others at a larger distance than the maximal distance they ought to be able to reach, and felt uncomfortable when others were below this limit (Figure 5). These findings are in line with previous observations showing that IPS is typically between 80 and 140 cm (Sorokowska et al., 2017), while PPS and PS are smaller (i.e., 50–70 cm; Ambrosini et al., 2012; Bourgeois et al., 2014; Holt et al., 2014; Iachini et al., 2016). The present study further highlights that PS was smaller than PPS. The difference was small though, explaining why some previous studies found that the presence of stimuli in PPS generates discomfort together with strong physiological responses (Kennedy et al., 2009; Cartaud et al., 2018, 2020; Ellena et al., 2020; Vieira et al., 2020). Despite the differences in average extent, the three spaces were positively correlated to each other, irrespectively of the gender of the facing virtual character and the presence of a face mask or not. This means that participants who had a large (or small) PPS representation were also those who preferred placing and tolerated others far away (or close). Moreover, PPS and IPS were commonly affected by gender, being shorter when participants interacted with female characters. It is worth noting that the effect of gender on PPS was only observed when the virtual characters were unmasked. We checked whether the effect of the character’s gender was similar depending on the gender of the participant and found an overall tendency for closer distances with female characters in both female and male participants (procedure and results reported in the Supplementary material). Thus, our results do not only replicate previous findings showing that action and social spaces are commonly affected by gender (Iachini et al., 2014, 2016), but go a step further by showing that they vary together across individuals, at least with virtual characters exhibiting a neutral facial expression. Altogether, these results support the idea that social spaces are rooted in the representation of PPS in relation to its sensorimotor nature, as indexed by the reachability judgments. Accordingly, it is likely that the sensorimotor representation of PPS serves as a spatial reference to specify interpersonal distances in social contexts (Coello and Cartaud, 2021).

Importantly, unmasked characters triggered discomfort already at further distances than masked characters, but face mask did not impact preferred inter-individual distance or reachability judgments at the group level (contrary to what has been previously observed; e.g., Cartaud et al., 2020). The effect of the face mask on both PS and IPS was, however, modulated by risk and germ aversion, with unmasked characters triggering more quickly discomfort and being preferentially placed further away in individuals who are risk and germ averse, while the reverse was observed in participants who are not averse to risk and germs. This supports the idea that the BIS affects inter-individual distances, though not necessarily in a direct way as shown by Hromatko et al. (2021), but also through the modulation of the effect of situational factors, such as the presence of a face mask, on these distances. The BIS is assumed to be triggered by perceptual cues connoting the presence of pathogens in the surrounding environment. These cues can also consist of conspecifics that behave in ways that increase the likelihood that infections will be spread by failing to observe the required sanitary practices. When detecting such cues, the BIS is assumed to react by triggering disgust and aversive cognition, as well as behavioral avoidance (Schaller, 2011). Hence, the presence of a face mask on certain virtual characters might certainly have cued the presence of pathogens in the environment, especially in a context where COVID-19 is still circulating. Thereby, it might have generated behavioral avoidance toward the characters that increased the risk of infection spreading, i.e., those who did not wear a face mask, especially in individuals with high risk and germ aversion, reflecting a reactive BIS. It is worth noting that we cannot exclude that the effects rather reflect approaching mechanisms toward masked characters. However, regarding the pathogens avoidance function of the BIS, it is more likely that high germ and risk aversion causes avoidance of people that are at risk than approach behaviors toward those that are not. The finding that individuals with low risk and germ aversion rather placed and tolerated unmasked characters closer than masked characters might be explained by a natural tendency toward gregariousness in individuals that do not perceive themselves as vulnerable to disease (Schaller, 2011). Nevertheless, these interpretations about how the BIS modulates the effect of the face mask are only speculative at this stage and would need further investigation to be specified.

It is worth underlining that, without considering the BIS, we only found an effect of face mask on PS, while it has been previously reported on both PS and IPS (Cartaud et al., 2020; Iachini et al., 2021; Lisi et al., 2021; Luckman et al., 2021; Kroczek et al., 2022). Moreover, we also failed to replicate the effect of face mask on perceived trustworthiness that was typically reported in these studies. One possible explanation is that the perception of face mask has changed since then. Indeed, the social and cultural meaning of face mask, and thereby the way they are perceived, have changed with their use and recommendation over the first months of the COVID-19 pandemic. For instance, from April to October 2020 face mask progressively switched from “symbol of disease” to “symbol of prevention” (Schönweitz et al., 2022). As most studies were conducted after the first months of the pandemic when face mask already reflected more prevention than disease, it is not surprising they found increased trust toward masked individuals. We collected our data more than two years after the beginning of the pandemic when face mask was not mandatory anymore and contaminations were in constant decrease. Hence, the face mask might only have remained a symbol of prevention to the participants with a reactive BIS. Accordingly, a recent study found only limited effects of face mask on first impressions of others (Twele et al., 2022).

The fact that the PPS was not affected by the face mask nor modulated by the BIS, while social spaces were, is in some aspect in line with the homeostatic theory of social interaction (Coello and Cartaud, 2021). As indicated by the authors, the IPS would be built on the basis of PPS representation with an extra margin that is flexible depending on the context. The authors suggested that this extra margin would adapt as a function of the perceived valence or threat of the social stimulus. Although we did not find evidence for a difference in terms of emotional valence between the masked and unmasked virtual characters, unmasked individuals usually represent a greater risk in terms of pathogen transmission (Aranguren, 2021). Accordingly, the extra margin may increase while interacting with unmasked individuals, in particular for participants showing a reactive BIS, leading to increased social distance while leaving unaffected PPS. The fact that face mask could be associated with risk compensation affecting social distances would require further investigations in the future.

To summarize, the present study highlighted the intrinsic relationship between action PPS and social PS and IPS. Furthermore, it confirmed the previous finding of reduced social spaces in the presence of individuals wearing a face mask. However, several years after the beginning of the pandemic, the effect was turned down probably due to habituation, so it was still observed in individuals characterized by high aversion to risk and germs. In conclusion, the present findings suggest that the regulation of the social spaces depends on the representation of PPS, but with an extra margin that is modulated by situational and personal factors in relation to the BIS.

## Data availability statement

The datasets presented in this study can be found in online repositories. The names of the repository/repositories and accession number(s) can be found below: https://osf.io/serjn/.

## Ethics statement

The studies involving human participants were reviewed and approved by the Research Ethics Board of the University of Lille. The patients/participants provided their written informed consent to participate in this study.

## Author contributions

LG collected and analyzed the data. Both authors contributed to the design of the study and the writing of the manuscript.

## Footnotes

1
https://osf.io/sp938
