# Supplementary material for: The influence of face mask on social spaces depends on the behavioral immune system
Source: Front Neurosci. 2022 Nov 11;16:991578. doi: 10.3389/fnins.2022.991578 (PMC9691846; doi:10.3389/fnins.2022.991578)
Supplement: Supplementary file 1 [file Data_Sheet_1.docx]

Supplementary Material

# Correlation between PPS, PS and IPS in each stimulus

All three spaces were positively correlated to each other in all stimuli. The Pearson *r* coefficients and the associated p-values as a function of the stimulus are reported in Supplementary Table 1.

|  | **Peripersonal** | | | | **Personal** | | **Interpersonal** | |
| --- | --- | --- | --- | --- | --- | --- | --- | --- |
|  | *r* | *p* | | *r* | | *p* | *r* | *p* |
| **Unmasked Male** |  | | |  | | |  | |
| **Peripersonal** | 1 | |  |  | | |  | |
| **Personal** | .52 | | <.001 | 1 | |  |  | |
| **Interpersonal** | .54 | | <.001 | .69 | | <.001 | 1 |  |
| **Unmasked Female** |  | |  |  | |  |  |  |
| **Peripersonal** | 1 | |  |  | |  |  |  |
| **Personal** | .51 | | <.001 | 1 | |  |  |  |
| **Interpersonal** | .55 | | <.001 | 0.63 | | <.001 | 1 |  |
| **Masked Male** |  | |  |  | |  |  |  |
| **Peripersonal** | 1 | |  |  | |  |  |  |
| **Personal** | .55 | | <.001 | 1 | |  |  |  |
| **Interpersonal** | .56 | | <.001 | .69 | | <.001 | 1 |  |
| **Masked Female** |  | |  |  | |  |  |  |
| **Peripersonal** | 1 | |  |  | |  |  |  |
| **Personal** | .52 | | <.001 | 1 | |  |  |  |
| **Interpersonal** | .60 | | <.001 | .68 | | <.001 | 1 |  |

**Supplementary Table 1.** Pearson correlation matrix for the average size of PPS, PS and IPS in each stimulus

# Effect of character’s gender across participant’s gender

We conducted exploratory analyses to investigate whether the effect of the character’s gender varies according to the gender of the participant. To do so, we split the data into males (N = 20) and females (N = 20) participants and conducted pairwise comparisons with Bonferroni correction between the extent of PPS/IPS/PS when facing male and female characters in each sample. Regarding IPS, the results showed that female participants preferentially placed female characters at a significantly shorter distance (125.4 ± 7.7 cm) than male characters (130.7 ± 7.8 cm), *t*(118) = -2.98, *p* = .003. While the same tendency was observed for male participants (M_females_ = 108.3 ± 6.4 cm, M_males_ = 111.2 ± 6.4 cm), the comparison failed to reach significance, *t*(118) = -1.58, *p* = .116. Regarding PS, the results showed that male participants tolerated female characters significantly closer (64.5 ± 6.2 cm) than male characters (68.4 ± 6.6 cm), *t*(118) = -2.62, *p* = .010, and that the same tendency was observed in female participants (M_females_ = 76.1 ± 6.7 cm, M_males_ = 77.3 ± 6.6 cm), even though not significant, *t*(118) = -0.78, *p* = .436. Finally, concerning PPS, the results showed that female participants judged female characters as reachable at significantly shorter distances (72.2 ± 4.0 cm) than male characters (74.5 ± 3.9 cm), *t*(118) = -1.98, *p* = .049, while a similar, but small non-significant tendency was observed in male participants (M_females_ = 82.9 ± 5.3 cm, M_males_ = 83.9 ± 5.3 cm). Hence, there seems to be an overall tendency for shorter distances when facing females than when facing males, however, the comparison failed to reach significance in some cases. However, this might be due to a lack of power associated with splitting the sample in two.
